# Depression and suicidality amongst infertile women: a hidden pandemic?

**DOI:** 10.1192/j.eurpsy.2022.484

**Published:** 2022-09-01

**Authors:** B. Ghosh Dastidar

**Affiliations:** Calcutta National Medical College, Psychiatry, Kolkata, India

**Keywords:** Infertility, Depression, suicide

## Abstract

**Introduction:**

The psychosocial impact of infertility has been well researched and documented. However very little research has been conducted to assess the causative relationship between infertility and serious psychiatric illness such as suicide.

We conducted a cross-sectional study to investigate suicidal risk ( suicidal ideation/ suicide attempts) amongst 100 infertile women undergoing infertility treatment at an IVF Centre based in Kolkata, India. Allied parameters such as depression, anxiety, quality of life were also studied.

**Objectives:**

The goal of the presence study was to assess the suicidal risk ( suicidal ideation / attempted suicide ) amongst infertile women undergoing infertility/ ART treatment. And to examine the possible etiological factors behind increased levels of suicidal risk amongst infertile women.

**Methods:**

Mini International Neuropsychiatric Interview was administered to 100 female patient’s undergoing IVF treatment and 100 control patient’s , visiting an IVF Centre based in Kolkata. Chi square test, independent t test and Z test used for statistical analysis.

**Results:**

According to the results obtained by assessment of MINI scale , the prevalence of major depressive disorder (50 % ) followed by Dysthymia (25%) was documented among infertile group while suicidality at 15% were significantly higher than other disorder (Z = 3.80, p> .001). No such cases of suicidality or Dysthymia was found among patients of control group. (fertile group).

**Conclusions:**

Routine screening of suicidal risk and depression should be conducted for all patients undergoing IVF treatment. Infertility specialists should recognize psychiatric morbidity amongst infertile patients for subsequent referral and treatment.

**Disclosure:**

No significant relationships.

